# Factors Influencing Nurses' Experiences and Support as Second Victims Following Adverse Events: A Scoping Review

**DOI:** 10.1002/nop2.70556

**Published:** 2026-08-13

**Authors:** Fang Yang, Xin Yi, Kejimu Sunzi, Jingru Zhou, Jiquan Zhang, Xiaoli Zhong, Xia Wu, Huawei Li

**Affiliations:** ^1^ Department of Pediatrics Deyang People's Hospital Deyang Sichuan China; ^2^ Department of Nursing Deyang People's Hospital Deyang Sichuan China; ^3^ Department of Gastroenterology Hospital of Chengdu University of Traditional Chinese Medicine Chengdu Sichuan China; ^4^ Nephrology Department Deyang People's Hospital Deyang Sichuan China; ^5^ Nephrology Department Affiliated Hospital of Nanjing University of Chinese Medicine Nanjing Jiangsu China

**Keywords:** adverse events, influencing factors, nurses, scoping review, second victims

## Abstract

**Aim:**

To analyse nurses' current status and influencing factors as second victims, experiencing and receiving support, to provide a scientific basis for future intervention research on nurses as second victims.

**Design:**

A scoping review.

**Methods:**

A scoping review was conducted using the framework by Arksey and O'Malley, systematically retrieving Chinese and English papers published up to March 27, 2024, from databases including CNKI, Wan Fang, VIP, China Biology Medicine Database, PubMed, Embase, Web of Science, CINAHL, Cochrane Library and Scopus. The search spanned from the inception of the databases to the specified date. Data were extracted and synthesized, presented in tabular form.

**Results:**

Twenty‐three studies were eventually included. After experiencing adverse events, nurses as second victims face significant distress, with varying perceptions of support. Different influencing factors, including sociodemographic, work, psychological and event‐related factors, were categorized and summarized, affecting the experience and support of nurses as second victims.

**Conclusion:**

Nurses who experience adverse events exhibit high levels of second‐victim distress and low perceived organizational support. Their experiences and support as second victims are influenced by sociodemographic, work‐related, psychological and adverse event factors. Future research should focus on longitudinal studies, multicultural and gender‐diverse designs, mixed‐methods research on high‐risk groups, and exploring interactions among influencing factors to improve support for second victims and enhance nursing quality and patient safety.

**Relevance to Clinical Practice:**

Nursing managers develop individualized support programmes for different influencing factors as well as the characteristics and needs of nurses' second victims and build a supportive nursing culture to mitigate the negative impact of adverse events on nurses, improve the quality of care and ensure patient safety.

**Patient or Public Contribution:**

No patient or public contribution.

## 1 Background

Patient safety has been a globally prioritized topic (Chan et al. 2018). No one should be harmed during a medical visit; patient safety is the cornerstone of quality care. However, as society advances, the complexity of medical work has led to the inevitability of medical adverse events, significantly impacting patient safety and care quality (Hall and Scott 2012).

Medical adverse events refer to any factors or incidents in clinical treatment and nursing activities that may affect the treatment outcomes, increase patient suffering and burden, potentially trigger medical disputes or accidents and impact the safety and well‐being of healthcare providers (Wei chao et al. 2009).

Among these, adverse events, medical errors and related incidents are among the main challenges to patient safety. Studies (Zhang et al. 2019) indicate that the incidence rate of medical adverse events ranges from 10.4% to 46.8%. In the United States, approximately 400,000 to 440,000 patients die annually due to medical adverse events, making it the third leading cause of patient deaths (Makary and Daniel 2016). Adverse events not only cause physical and emotional trauma to patients but also impose psychological burdens on healthcare providers, as evidenced by studies showing that second victims often experience anxiety, depression and burnout (Busch et al. 2020). In adverse events, patients are the direct victims, or the first victims, while healthcare providers are the second victims.

Professor Wu (2000) introduced the concept of ‘second victims’ in 2000, but the term was not clearly defined then, only used to describe healthcare professionals affected by adverse events in medical incidents. In 2009, Scott et al. (2009) formally defined the concept of second victims as healthcare providers who participated in unexpected patient adverse events, medical errors and/or injuries related to patients and suffered trauma and harm related to the event. More recently, an evidence‐ and consensus‐based definition has been proposed, describing the second victim as ‘any healthcare worker, directly or indirectly involved in an unanticipated adverse patient event, unintentional healthcare error or patient injury, who becomes victimized in the sense that the worker is also negatively impacted’ (Vanhaecht et al. 2022). This definition broadens the scope to include all healthcare workers and emphasizes the negative impact on the individual, which is particularly relevant for understanding nurses' experiences. After adverse events occur, most hospital administrators focus on the causes, handling strategies and prevention, often overlooking the recognition and support for second victims. This is consistent with the Psychological Safety Theory, suggesting that the second victim phenomenon frequently occurs in environments lacking trust and open communication. Without adequate support, nurses may internalize blame, leading to long‐term psychological distress (Edmondson 1999; Kahn 1990).

Research shows that second victims may experience anxiety, fear, increased breathing rate, insomnia and a range of psychological, physiological and occupational second‐victim syndromes (Chan et al. 2017; Marmon and Heiss 2015; Tamburri 2017). A study found that 49.7% of healthcare professionals had experienced patient safety incidents and 66.0% of them developed anxiety and depression, worrying about their ability to fulfil their professional duties (Stone 2020). Multiple studies have shown that over 50% of nurses who have experienced adverse events report psychological trauma, severe insomnia, guilt, self‐blame, intrusive reflections, colleague bias and discrimination, occupational burnout and even career crises (Chan et al. 2018; Bohomol 2019; Burlison et al. 2021; Lewis et al. 2013). The psychological trauma from adverse events is persistent, potentially leading to sleep disorders and mood disorders, and significantly impacting the physical and mental health and safety of nursing care (Wu 2000). Busch et al. (2020) found that over two‐thirds of second victims suffer from long‐term distress, anxiety, anger, regret and physical and emotional issues.

All healthcare professionals can become second victims, but nurses are at a higher risk due to factors such as high workload, inadequate staffing and longer patient contact time. Additionally, nurses are integral to patient safety care, making them a high‐risk group for being second victims (Chan et al. 2017). As society evolves, the demand for nursing care increases, and nurses face heavy workloads, making them at high risk for medical adverse events. From the perspective of Psychological Safety Theory (Edmondson 1999; Kahn 1990), as frontline workers, nurses in environments lacking psychological safety may fear being blamed for disclosing errors, leading to emotional suppression and prolonged ‘second victim’ trauma. Additionally, the Conservation of Resources theory (COR) (Hobfoll 1989) further explains that high workloads and insufficient organizational support deplete nurses' psychological resources, making them more vulnerable to long‐term suffering as second victims and hindering their recovery from adverse events. Therefore, it is necessary to focus on the group of nurses who are second victims.

This study conducts an in‐depth exploration based on the Psychological Safety Theory and the Conservation of Resources (COR) Theory. The Psychological Safety Theory (Edmondson 1999; Kahn 1990) indicates that in a team environment, if individuals fail to perceive a sense of safety where expressing their ideas, admitting mistakes or seeking help will not be punished or denied, they will inhibit communication behaviours, thereby hindering problem‐solving and the learning process. In a medical setting, if the hospital culture lacks a psychologically safe atmosphere, nurses may be afraid to report errors or seek help after adverse events due to fear of blame, leading to the continuous accumulation of negative emotions and further exacerbating their psychological trauma. The Conservation of Resources Theory (Hobfoll 1989) holds that individuals have an instinct to protect and accumulate resources. When facing stressors, if the rate of resource depletion exceeds the rate of replenishment, individuals will fall into a state of resource exhaustion, leading to negative outcomes such as stress and anxiety. For nurses, stressors such as long‐term high‐intensity work and low‐level organizational support will continuously consume their psychological resources, making it more difficult for them to recover after experiencing adverse events.

Currently, scholars have investigated the experiences of second victims and factors influencing support, yet research specific to nurses lacks systematic and comprehensive synthesis. Given the critical role of hospital nursing and the potential risks of adverse events to nurses' mental health, this study employs the scoping review framework proposed by Arksey et al. (Arksey and O'Malley 2005), grounded in Psychological Safety Theory (Edmondson 1999; Kahn 1990) and Conservation of Resources Theory (Hobfoll 1989). It synthesizes studies on nurses' experiences as second victims and associated support factors, aiming to provide a scientific foundation for developing intervention strategies and constructing support systems through theoretical integration.

## 2 Methods

A scoping review is defined as a form of knowledge synthesis that systematically searches, selects and integrates existing knowledge to address exploratory research, aiming to map key concepts, evidence types and research gaps related to a defined field or area (Colquhoun et al. 2014). Scoping reviews are useful for examining emerging evidence, identifying knowledge gaps and providing a rigourous and transparent method for recognizing and mapping existing evidence (Arksey and O'Malley 2005). This scoping review was conducted based on the methodology framework by Arksey and O'Malley (Arksey and O'Malley 2005), encompassing five stages: defining the research question, identifying relevant studies, study selection, charting the data and summarizing, synthesizing and reporting the results. The PRISMA checklist for scoping reviews guided this review, enhancing the rigour of the reporting (Tricco et al. 2018). A review programme was developed for this study, but the research programme was not registered. Additionally, to minimize the potential for publication bias, we conducted a search of grey literature sources including dissertations, conference proceedings and unpublished reports. However, no additional records meeting the inclusion criteria were identified from these sources. We also attempted to contact authors of relevant studies to obtain any unpublished data or additional information, but none were obtained.

### 2.1 Review Question

This scoping review aimed to analyse the current state of experiences and support for nurses as second victims, along with the factors influencing these experiences. The review question is as follows:

What is known in the literature about nurses as second victims following adverse events?

### 2.2 Inclusion and Exclusion Criteria

Based on the PCC principles (Lockwood et al. 2019), the criteria for inclusion are as follows:

1. Participants (P): Registered nurses engaged in clinical care who are second victims of medical adverse events. This study was based on an understanding of the second victim and included subjects that encompassed all nurses who may have been involved in an adverse event, including but not limited to nurses who self‐reported as second victims. Our goal was to focus on nurses who experienced adverse events and were adversely affected as a result.
2. Concept (C): Original research involving the impact factors of nurses' experiences and support as second victims. The types of literature included in this study included all types of research designs. Included studies could be validated scales (e.g., Second Victim Experience and Support Tool) assessing nurses' experience and level of support as a second victim of an adverse event, qualitative studies, quantitative studies, mixed studies and so on. The concept addressed in this study is ‘second victim’. For this scoping review, a second victim is defined as a healthcare provider who has experienced an unanticipated adverse patient event, medical error and/or patient‐related injury and who, as a result, has suffered trauma and harm related to the event.
3. Context (C): This review includes all types of clinical care settings, including psychiatric wards, emergency departments, operating rooms, paediatrics and hospital units. The goal of this study was to gain a comprehensive understanding of nurses as secondary victims in different care settings.

The exclusion criteria are as follows:

1. Literature without full‐text access, conference papers.
2. Duplicate publications.
3. Non‐Chinese and English literature
4. Cases, review literature, editorials, conference abstracts, letters to the editor, opinion pieces;
5. In the literature, the study was conducted on non‐nursing staff such as trainee nursing students, doctors, pharmacists, etc.

### 2.3 Search Strategy

A comprehensive search was conducted across 10 databases (CNKI, Wan fang, VIP, CBM, PubMed, Embase, Web of Science, CINAHL, Cochrane Library, Scopus) from the inception of each database to March 27, 2024. The search terms included: Nurs*, Nurs* Personnel, Registered Nurs*, Health Personnel, Health Care Provider*, Health Care Professional*, Nurs* Practitioner*, second victim*, Second victim* Syndrome, Second victim* phenomenon. The search strategy used was MeSH terminology and keywords to identify all relevant studies on the topic. The process is as follows:

1. Nurs* OR Nurs* Personnel OR Registered Nurs* OR Health Personnel OR Health Care Provider* OR Health Care Professional* OR Nurs* Practitioner*.
2. Second victim* OR Second victim* Syndrome OR Second victim* phenomenon.
3. 1 AND 2.

In this study, specific search strategies were adopted for each database. We modified the keywords and subject headings to suit the requirements of the databases. Keywords using the truncation symbol (*) to broaden search terms within title and abstract fields were used in an initial search. In addition, Boolean operators or connecting words ‘OR’ and ‘AND’ were used to limit, broaden or define searches within and between categories.

While the selected databases cover mainstream academic resources in the fields of nursing, medicine, psychology and other related disciplines, non‐English and non‐Chinese literature was not included. This limitation may underestimate culturally specific factors and affect the global representativeness of the results. This limitation is primarily due to the language capabilities of the research team, but it also provides a direction for future cross‐cultural comparative studies. The full search strategies for each database, including search terms, Boolean operators and field limits, are provided in Data S1.

### 2.4 Literature Screening and Data Extraction

The retrieved literature was imported into Endnote 20 software to screen and remove duplicates. The literature was independently screened by two researchers (XW and KjmSZ) based on predefined inclusion and exclusion criteria. Each researcher independently reviewed the title and abstract to perform the primary screening and then read the full text to perform the secondary screening. In instances of disagreement during the screening process, a third researcher (JqZ) was consulted to facilitate a discussion and assist in resolving the disagreement, thereby ensuring the selection of literature that met the established criteria. All researchers were trained in evidence‐based methods. Two researchers (XW and KjmSZ) independently extracted data from the included studies using a pre‐designed Excel spreadsheet. For studies with inconsistent extraction results, a third researcher (JqZ) organized discussions or consultations to resolve discrepancies. To ensure the effectiveness of data extraction, the two researchers initially pre‐extracted data from five studies to standardize the understanding of data fields and adjusted the extraction form before formally extracting data. Although we did not perform formal inter‐rater reliability statistics (such as Cohen's kappa), to ensure consistency, we assessed inter‐rater reliability during the full‐text screening and data extraction phases by conducting dual cross‐checks and expert arbitration to ensure the accuracy and consistency of data extraction.

The specific data points collected included:

1. Study Characteristics: Author, publication year, country of study, research purpose, study type, sample size and characteristics of the nurse population.
2. Influencing Factors: Sociodemographic factors (age, gender, marital status, income, education level), work‐related factors (job title, years of work experience, employment status, number of night shifts, department, hospital level, perceived leadership support, perceived organizational mismanagement, insufficient information, cultural barriers and perceived organizational climate and support), psychological factors (psychological resilience, psychological hardiness, coping level, professional quality of life, participation in psychological training) and adverse event‐related factors (direct experience of patient safety incidents, frequency of adverse events experienced, severity of adverse events, frequency of adverse event reporting, participation in department‐level adverse event discussions and perceived level of patient safety culture).
3. Data Collection Tools: SVEST scale.
4. Main Findings and Conclusions: Summary of the main results and significance of each study.

## 3 Results

### 3.1 Study Characteristics

**FIGURE 1:**
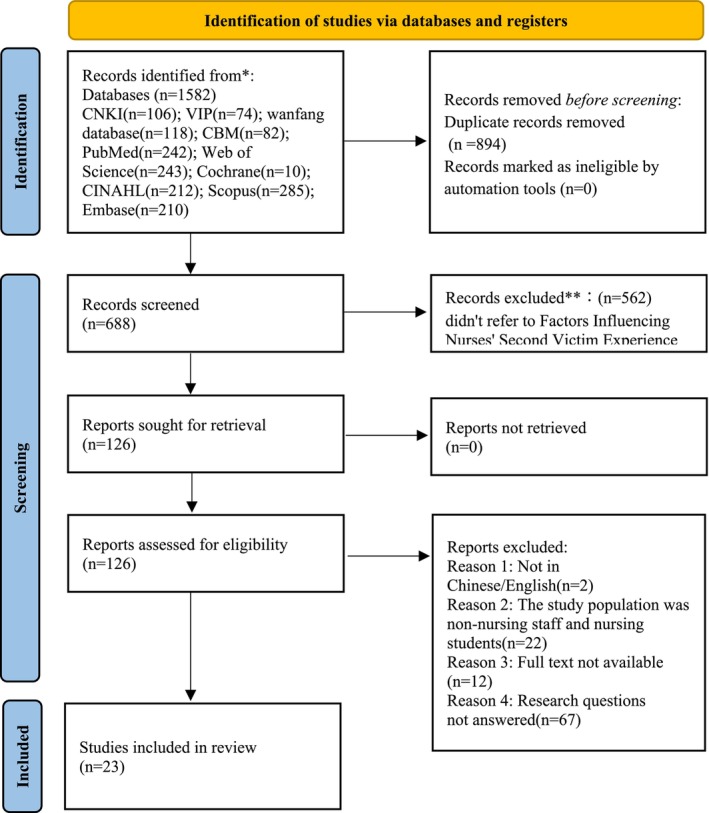
PRISMA 2020 flow diagram.

A total of 1582 studies were obtained from this scoping review search, with 688 remaining after removal of duplicates using Endnote20. Following a thorough examination of the article titles and abstracts, 562 were excluded from the study, leaving 126 for further consideration. Following a thorough examination of the full texts, 103 studies were excluded for various reasons, including the unavailability of the full text, incomplete information or a lack of relevance to the study's topic. Ultimately, 23 studies were deemed suitable for inclusion in the final analysis. Among these, 21 studies employed cross‐sectional designs (Guiru et al. 2020; Guiru, Huimin, Rongrong, Shihua, et al. 2019; Chen et al. 2020; Linxia et al. 2021; Jie et al. 2020; Huijie et al. 2023; Huang et al. 2022; Zixia et al. 2021; Peitao et al. 2021; Zuokun et al. 2023; Anfang et al. 2022; Sihan et al. 2023; Mingdan et al. 2022; Mok et al. 2020; Xiao et al. 2022; Wang et al. 2023; Jing et al. 2020; Hongning et al. 2021; Yan et al. 2022; Ruixin et al. 2022; Houqin et al. 2023), 1 qualitative design (Mokhtari et al. 2018) and 1 mixed‐methods design (Tang et al. 2024). All studies were published between 2018 and 2024. Twenty‐one studies were conducted in China, one in Singapore, and one in Iran. The study participants were all nurses, and female nurses constituted the majority across all included studies. This demographic profile aligns with the gender distribution characteristics of the global nursing profession (World Health Organization 2020). The PRISMA 2020 flow diagram (Figure 1) illustrates the search process, and Table 1 provides detailed information on the included articles.

**TABLE 1:** Basic information about the included studies.

Author(s), year, country	Aim	Study design	Sample size (male/female)	Influencing factors	Research tool
Chen et al. (Guiru et al. 2020), China	To explore the relationship between support for nurses as second victims and hospital patient safety culture, and to provide reference for the second victim intervention.	Cross‐sectional study	12/2775	Frequency of adverse event reporting perceived patient safety culture	Guiru Chen Chinese version SVEST
Chen et al. (Guiru, Huimin, Rongrong, Shihua, et al. 2019), China	To investigate the experiences of the nurses as the second victims after the adverse events.	Cross‐sectional study	3/255	Age, job title, working experience, marital status, participation in adverse event discussions at the nursing department level, type of adverse event	Guiru Chen Chinese version SVEST
Chen et al. (2020), China	To investigate the status of experience and support of nurses as the second victims in patient safety incidents in Chongqing, and to analyse its relevant factors.	Cross‐sectional study	47/1375	Job title, age, marital status, income	Jiaojiao Chen Chinese version SVEST
Chen et al. (Linxia et al. 2021), China	To understand the nurses' experience and support status as the second victim in psychiatric patient safety incidents and analyse the related influencing factors.	Cross‐sectional study	18/202	marital status, working experience, Level of leadership support for work, coping style	Jiaojiao Chen Chinese version SVEST
Deng et al. (Jie et al. 2020), China	To explore the impact of organizational atmosphere perception on the second victim's experience and support.	Cross‐sectional study	17/332	Level of hospital, Organizational Climate Perception	Jiaojiao Chen Chinese version SVEST
Hou et al. (Huijie et al. 2023), China	To understand the experience and support status of oncology nurses as second victims of adverse events, and to explore its influencing factors, to provide reference for nursing intervention for second victims of adverse events.	Cross‐sectional study	81/2458	Age, job title, salary, working experience, department, number of adverse events in career	Jiaojiao Chen Chinese version SVEST
Huang et al. (2022), China	To investigate the experience and support of nurses as second victims in adverse events and explore factors.	Cross‐sectional study	12/2775	Age, sex, adverse event level	Guiru Chen Chinese version SVEST
Kuang et al. (Zixia et al. 2021), China	To investigate the experience and supporting status of Intensive Care Unit (ICU) nurses as the second victims, and to provide support for the group.	Cross‐sectional study	28/243	Academic qualifications, staffing status, years of experience, hospital grade, number of night shifts	Guiru Chen Chinese version SVEST
Li et al. (Peitao et al. 2021), China	To investigate the status of second victim effects of nurses in tertiary hospitals after adverse events and analyse its influencing factors.	Cross‐sectional study	813MF	Department, job title, marital status, nature of employment, classification and type of adverse events	Jiaojiao Chen Chinese version SVEST
Li et al. (Zuokun et al. 2023), China	Understanding ICU nurses' internal experience and support needs after an adverse event and analysing the impact of a sense of organizational support and psychological resilience on ICU nurses' experience and support as second victims.	Cross‐sectional study	56/372	Working experience, Whether or not they experience analytical discussion debriefing at the nursing department level, perceived organizational support, psychological resilience	Guiru Chen Chinese version SVEST
Liu et al. (Anfang et al. 2022), China	To investigate the current status of second‐victim experiences and support for low‐status nurses and analyse influencing factors.	Cross‐sectional study	289F	Department, form of employment, marital status, number of adverse events in career experience	Jiaojiao Chen Chinese version SVEST
Liu et al. (Sihan et al. 2023), China	To understand the current situation of nurses' experience and support as the second victim of adverse nursing events and analyse the influencing factors.	Cross‐sectional study	44/377	Job title, education level, Knowledge of the concept of ‘second victim’, Number of night shifts, psychological resilience	Jiaojiao Chen Chinese version SVEST
Ma et al. (Mingdan et al. 2022), China	To explore the impact of resourcefulness on second victim experience and support of emergency department (ED) nurses.	Cross‐sectional study	89/330	Resourcefulness level, Job satisfaction, Number of night shifts	
Mok et al. (2020), Singapore	The study aimed to investigate nurses' second victim experience and quality of support resources in Singapore.	Cross‐sectional study	11/1049	Age, working experience	
Mokhtari et al. (2018), Iran	The current study was conducted to identify the barriers regarding supporting nurses as second victims of nursing errors in clinical settings in Iran.	Qualitative study	4/14	Mismanagement, Cultural barriers, inadequate information, Legal barriers	
Song et al. (Xiao et al. 2022), China	To investigate nurses' experience and support status in psychiatric hospitals as second victims in patient safety incidents and analyse the influencing factors.	Cross‐sectional study	77/203	Age, working experience, direct experience with patient safety events	
Tang et al. (2024), China	To investigate the current status of experience and support of nurses as second victims and explore its related factors in nurses.	Mixed Study	406MF	Gender, age, income, job title, Education, marital status, working experience	
Wang et al. (2023), China	To understand the experience and support status of psychiatric nurses as the second victim in adverse events and analyse the factors affecting the experience and support of their second victim.	Cross‐sectional study	38/1895	Gender, hospital level, working experience, shifts, psychological resilience	
Wei et al. (Jing et al. 2020), China	To investigate paediatric nurses' experience and support status as second victims and to analyse the influencing factors.	Cross‐sectional study	4/217	Department, working experience, number of adverse events in career experience	
Xu et al. (Hongning et al. 2021), China	To explore the experience and support level of nurses as second victims, to probe the association between support and professional quality of life, and to provide a basis for the formulation of nursing intervention for second victims.	Cross‐sectional study	10/411	Professional quality of life	
Yan et al. (2022), China	To describe the experience and support of Chinese healthcare professionals as second victims of PSIs.	Cross‐sectional study	17/1185	Sex, age, job title, job position	
Zhang et al. (Ruixin et al. 2022), China	To investigate nurses' experience and support status as the second victims of medical adverse events and analyse the influencing factors.	Cross‐sectional study	15/211	Age, marital status, job title, working experience, form of employment, income, whether psychological training has been conducted	
Zong et al. (Houqin et al. 2023), China	To understand the current status of surgical nurses' experience and support for being a second victim of an adverse event and to analyse the factors that influence it.	Cross‐sectional study	29/150	Sex, age, working experience, marital status	

*Note:* ‘—’ indicates not mentioned in the text; ‘SVEST’ is the Second Victim Experience and Support Tool; the symbol ‘F’ stands for female and the symbol ‘MF’ indicates that the number of women and men is not specified in the text.

### 3.2 Factors Influencing Nurses' Experiences and Support as Second Victims

This scoping review analysed the 23 papers included in the literature and found that various factors influence nurses' second‐victim experience and level of support as second victims of adverse events. We categorized the identified influences into four areas: sociodemographic, work, psychological and adverse event factors. Firstly, socio‐demographic factors encompass age, gender, marital status, income and education. Secondly, work factors include job title, working experience, the form of employment, number of night shifts, department, hospital level, degree of leadership support for the work, mismanagement, cultural barriers, inadequate information, legal barriers, organizational climate perception and perceived organizational support. Thirdly, psychological factors include psychological resilience, mental toughness, resourcefulness level, quality of professional life and development of psychological training. Psychological resilience, mental fortitude, resourcefulness level, quality of professional life and development of psychological training; and Adverse event factors: direct experience of patient safety events, number of adverse events experienced, grading of adverse events, frequency of adverse event reporting, participation in adverse event discussions at the nursing department level, and level of awareness of patient safety culture.

These categorizations reflect the complexity of the nurse's experience and support as a second victim and how multiple factors intersect to influence an individual's response and recovery.

### 3.3 Current Status of the Second Victim Experience and Support for Nurses

According to the findings of the included studies, nurses as second victims in the context of adverse events experience high levels of distress (Guiru, Huimin, Rongrong, Shihua, et al. 2019; Chen et al. 2020; Linxia et al. 2021; Jie et al. 2020; Huang et al. 2022; Zixia et al. 2021; Peitao et al. 2021; Zuokun et al. 2023; Anfang et al. 2022; Sihan et al. 2023; Mingdan et al. 2022; Mok et al. 2020; Xiao et al. 2022; Wang et al. 2023; Jing et al. 2020; Hongning et al. 2021; Ruixin et al. 2022; Houqin et al. 2023) and psychological distress (Guiru, Huimin, Rongrong, Shihua, et al. 2019; Chen et al. 2020; Linxia et al. 2021; Jie et al. 2020; Huang et al. 2022; Zixia et al. 2021; Peitao et al. 2021; Zuokun et al. 2023; Anfang et al. 2022; Mingdan et al. 2022; Xiao et al. 2022; Tang et al. 2024; Wang et al. 2023; Hongning et al. 2021; Yan et al. 2022; Ruixin et al. 2022; Houqin et al. 2023). There is a notable variation in the perceived support received by second victims. Four studies (Jie et al. 2020; Tang et al. 2024; Wang et al. 2023; Yan et al. 2022) highlight the predominance of management support, six studies (Chen et al. 2020; Jie et al. 2020; Wang et al. 2023; Hongning et al. 2021; Yan et al. 2022; Houqin et al. 2023) emphasize colleague support and four studies (Linxia et al. 2021; Anfang et al. 2022; Mingdan et al. 2022; Xiao et al. 2022) underscore the importance of family and friend support. Conversely, ten studies (Guiru et al. 2020; Guiru, Huimin, Rongrong, Shihua, et al. 2019; Jie et al. 2020; Zuokun et al. 2023; Anfang et al. 2022; Sihan et al. 2023; Xiao et al. 2022; Jing et al. 2020; Ruixin et al. 2022) indicate that second victims receive less support from colleagues and management. Six studies (Guiru et al. 2020; Jie et al. 2020; Zuokun et al. 2023; Wang et al. 2023; Ruixin et al. 2022; Houqin et al. 2023) report lower levels of support from family and friends.

### 3.4 Assessment Tools for the Second Victim Experience and Support

The assessment of the second victim experience and support for nurses primarily relies on the Second Victim Experience and Support Tool (SVEST). This tool, developed by American scholars Burlison et al. (2017), comprises seven dimensions (psychological distress, physical distress, colleague support, institutional support, superior support, family and friend support and occupational self‐awareness) and two outcome variables (absenteeism, intention to leave), totaling 29 items. The Cronbach's α coefficients for the dimensions range from 0.61 to 0.89. The tool employs a Likert 5‐point rating scale, with a total score ranging from 29 to 145. A higher SVEST score indicates greater distress and lower levels of support experienced by second victims. Currently tested cross‐culturally in countries around the world, the scale is widely used to assess the level of experience and support for second victims. From the literature included in this study, it was found that scholars Guiru, Huimin, Rongrong, Xiaoli, and Shao (2019) and Chen (2020) have translated and validated the SVEST. The former retained the original scale's dimensions, with a Cronbach's α coefficient of 0.892. The latter modified the wording of the scale items, resulting in a final version with six dimensions and 24 items, with a Cronbach's α coefficient of 0.824. Both versions demonstrate good reliability and validity, suitable for evaluating the experience and support of nurses as second victims. Both versions demonstrate good reliability and validity, suitable for evaluating the experience and support of nurses as second victims. In this study, eight articles used the version translated by Guiru, Huimin, Rongrong, Xiaoli, and Shao (2019), fourteen articles used the version translated by Chen (2020), and one article used the version developed by Burlison et al. (2017).

### 3.5 Influencing Factors on the Second Victim Experience and Support for Nurses

#### 3.5.1 Sociodemographic Factors

Studies have found that older (Tang et al. 2024), male nurses (Huang et al. 2022), those who are married (Peitao et al. 2021; Anfang et al. 2022; Houqin et al. 2023), and those with higher income (Chen et al. 2020; Ruixin et al. 2022) experience higher levels of distress as second victims and have a stronger intention to leave their jobs. Conversely, younger (Mok et al. 2020; Xiao et al. 2022; Tang et al. 2024; Ruixin et al. 2022; Houqin et al. 2023), female nurses (Tang et al. 2024), those who are unmarried or divorced (Linxia et al. 2021; Tang et al. 2024; Ruixin et al. 2022), those with lower income (Busch et al. 2020), and those with lower educational attainment (Sihan et al. 2023; Tang et al. 2024) may experience more severe distress and lower occupational self‐efficacy. Moreover, older (Guiru, Huimin, Rongrong, Shihua, et al. 2019; Chen et al. 2020; Linxia et al. 2021; Tang et al. 2024; Houqin et al. 2023), married nurses (Guiru, Huimin, Rongrong, Shihua, et al. 2019; Chen et al. 2020; Linxia et al. 2021; Tang et al. 2024) may receive higher levels of support from family, friends (Guiru, Huimin, Rongrong, Shihua, et al. 2019; Huijie et al. 2023; Xiao et al. 2022) and colleagues (Chen et al. 2020; Linxia et al. 2021; Tang et al. 2024; Houqin et al. 2023) after experiencing adverse events. On the other hand, male nurses (Huang et al. 2022; Tang et al. 2024; Wang et al. 2023; Yan et al. 2022), those with lower income (Tang et al. 2024), and those with higher educational attainment (Zixia et al. 2021) may receive lower levels of support from family and friends and the organization.

#### 3.5.2 Work Factors

Research indicates that nurses with higher professional ranks (Guiru, Huimin, Rongrong, Shihua, et al. 2019; Huijie et al. 2023; Peitao et al. 2021; Sihan et al. 2023; Yan et al. 2022), longer years of service (Linxia et al. 2021; Zuokun et al. 2023; Xiao et al. 2022; Wang et al. 2023; Houqin et al. 2023), contract‐based employment (Peitao et al. 2021; Anfang et al. 2022; Ruixin et al. 2022), frequent night shifts (Zixia et al. 2021; Sihan et al. 2023; Mingdan et al. 2022; Wang et al. 2023), lower job satisfaction (Mingdan et al. 2022) and a better understanding of the ‘second victim’ concept (Sihan et al. 2023) experience more distress after adverse events and have a stronger intention to leave. Other studies suggest that nurses with lower professional ranks (Chen et al. 2020; Tang et al. 2024; Ruixin et al. 2022), shorter years of service (Mok et al. 2020; Tang et al. 2024; Jing et al. 2020; Ruixin et al. 2022), and in‐service status (Zixia et al. 2021; Houqin et al. 2023) may experience higher levels of distress than second victims. The impact of adverse events on nurses varies by department (Huijie et al. 2023), with emergency department (Peitao et al. 2021; Anfang et al. 2022) and intensive care unit nurses (Jing et al. 2020) experiencing more severe trauma. Additionally, nurses with higher professional ranks (Guiru, Huimin, Rongrong, Shihua, et al. 2019; Huijie et al. 2023; Sihan et al. 2023; Yan et al. 2022), longer years of service (Guiru, Huimin, Rongrong, Shihua, et al. 2019; Linxia et al. 2021; Huijie et al. 2023; Jing et al. 2020), higher hospital levels (Jie et al. 2020; Zixia et al. 2021; Wang et al. 2023), frequent night shifts (Zixia et al. 2021), in‐service status (Houqin et al. 2023), better organizational atmosphere perception (Jie et al. 2020), leadership support and organizational support may receive more support after experiencing adverse events. However, nurses with frequent night shifts (Sihan et al. 2023; Mingdan et al. 2022) and in‐service status (Zixia et al. 2021) may receive less support. Furthermore, the management of the organization, lack of information about the second victim phenomenon, cultural barriers (Mokhtari et al. 2018), leadership support for work (Linxia et al. 2021) and organizational support (Zuokun et al. 2023) can influence support for second victims.

#### 3.5.3 Psychological Factors

Studies have found that nurses with better professional quality of life (Hongning et al. 2021), higher psychological resilience (Zuokun et al. 2023), higher psychological toughness (Sihan et al. 2023; Wang et al. 2023), higher intelligence quotient (Mingdan et al. 2022), and those who have undergone psychological training (Ruixin et al. 2022) experience less distress as second victims, have a lower tendency to be absent or leave, and receive higher levels of support.

#### 3.5.4 Adverse Event Factors

Research indicates that nurses who directly experience patient safety incidents (Xiao et al. 2022), those who experience multiple adverse events (Huijie et al. 2023; Anfang et al. 2022; Jing et al. 2020), those who experience adverse events of higher severity (Huang et al. 2022; Peitao et al. 2021), and those who participate in nursing department‐level adverse event discussions (Guiru, Huimin, Rongrong, Shihua, et al. 2019) experience higher levels of distress as second victims, leading to feelings of burnout and a desire to leave. Conversely, studies (Zuokun et al. 2023) suggest that nurses who do not participate in nursing department‐level adverse event discussions experience higher levels of psychological distress. The more positive the approach to dealing with adverse events (Linxia et al. 2021), the higher the level of awareness of patient safety culture, and the higher the frequency of adverse event reporting (Guiru et al. 2020), the higher the level of support received by nurses.

## 4 Discussion

### 4.1 Attention to the Current State of Nurses' Experiences and Support as Second Victims

The review highlights the intense psychological and physical stress responses and job insecurity experienced by nurses as second victims in adverse events. The reasons for this include post‐event concerns over patient prognosis, the emergence of negative emotions such as anxiety and fear, a decline in self‐identification, lack of understanding from patients or their families, and blame from managers (Delacroix 2017). Severe psychological distress can lead to burnout and a sense of low self‐worth, increasing the risk of clinical accidents and patient safety issues. Therefore, it is crucial to pay attention to the negative emotions of nurses in adverse events, assess their mental state, understand the causes of the second victim phenomenon, and help them restore their professional self‐efficacy and psychological resilience to alleviate physical and mental distress. Moreover, the support received by nurses as second victims varies, with a lower level of organizational support noted. This can be attributed to factors such as punitive measures from managers and colleagues (Zuokun et al. 2023), a lack of understanding and concern for second victims (Guiru, Huimin, Rongrong, Shihua, et al. 2019; Linxia et al. 2021; Zuokun et al. 2023), and a focus on the impact of adverse events on patients rather than on the nurses themselves (Zixia et al. 2021; Sihan et al. 2023). This is consistent with the psychological safety theory, which posits that a lack of interpersonal acceptance within healthcare teams can hinder nurses from seeking support following adverse events (Edmondson 1999). Additionally, nurses who are in a punitive and blame‐oriented culture are less likely to seek organizational support, thereby exacerbating feelings of guilt and isolation (Kahn 1990). Studies indicate that second victims have a high demand for organizational support (Edrees and Wu 2021). Effective organizational support can serve as a strong pillar for second victims, reducing their distress and aiding in their recovery from trauma (Tang et al. 2024; Kim et al. 2020). Thus, nursing administrators should accurately assess the current state of support received by second victims, provide targeted strategies and support based on their needs and expectations, enhance organizational support and alleviate their distress.

### 4.2 Importance of the Second Victim Experience and Support Scale

This review found that the Second Victim Experience and Support Scale is important in three ways. Firstly, to quantify and identify the psychological and emotional impact of nurses after experiencing an adverse event and to assess the level of psychological distress in nurses. Nurses may experience psychological distress and emotional trauma as a second victim of an adverse event (Guiru, Huimin, Rongrong, Shihua, et al. 2019; Chen et al. 2020; Linxia et al. 2021; Jie et al. 2020; Huang et al. 2022; Zixia et al. 2021; Peitao et al. 2021; Zuokun et al. 2023; Anfang et al. 2022; Mingdan et al. 2022; Xiao et al. 2022; Tang et al. 2024; Wang et al. 2023; Hongning et al. 2021; Ruixin et al. 2022; Houqin et al. 2023), and the scale identifies the degree of psychological distress of the second victim and helps nursing administrators to more accurately understand the nurse's experience of being a second victim, thus providing a basis for providing appropriate support and intervention. Second, identifying nurses' support needs after experiencing an adverse event is critical to building an effective support system. This review found that nurses perceived a low level of organization as second victims (Guiru et al. 2020; Guiru, Huimin, Rongrong, Shihua, et al. 2019; Linxia et al. 2021; Zixia et al. 2021; Zuokun et al. 2023; Anfang et al. 2022; Sihan et al. 2023; Xiao et al. 2022; Jing et al. 2020; Ruixin et al. 2022), suggesting that this is an area that requires attention and improvement by nursing managers. Therefore, nursing administrators should enhance support for nurses ‘second victims and construct effective interventions to reduce nurses’ psychological stress and burnout. Finally, the scale helps to analyse the factors that influence the experience and level of support for nurses' second victims, such as job income (Chen et al. 2020; Tang et al. 2024; Ruixin et al. 2022), job title (Guiru, Huimin, Rongrong, Shihua, et al. 2019; Chen et al. 2020; Huijie et al. 2023; Peitao et al. 2021; Sihan et al. 2023; Yan et al. 2022; Ruixin et al. 2022), age (Guiru, Huimin, Rongrong, Shihua, et al. 2019; Linxia et al. 2021; Huijie et al. 2023; Tang et al. 2024; Yan et al. 2022; Houqin et al. 2023) and marital status (Guiru, Huimin, Rongrong, Shihua, et al. 2019; Chen et al. 2020; Linxia et al. 2021; Tang et al. 2024; Ruixin et al. 2022). This information provides a scientific basis for care managers to develop effective interventions and build support systems. At the same time, it helps to improve nursing practice and the culture of patient safety, thus protecting the well‐being of nurses, improving the quality of care and ensuring patient safety.

### 4.3 Attention to the Male Nurse Second Victim Group

The review reveals that gender can influence the experience and support levels of nurses as second victims (Huang et al. 2022; Tang et al. 2024; Wang et al. 2023; Yan et al. 2022). Male nurses, in particular, experience higher levels of distress and a stronger sense of professional failure, with lower levels of support. Under the influence of cultural and societal factors, male nurses face multiple pressures and challenges when experiencing adverse events, leading to severe psychological and physical distress and a low sense of professional gain. At present, nursing managers' attitudes towards second victims in various hospitals are influenced by traditional concepts, and there is a general tendency to criticize and punish nurses after adverse events (Yongli et al. 2019), thereby exacerbating the second victim experience of male nurses. Hospital institutions' punishment and accountability systems fail to understand male nurses, and societal discourse reinforces their inner trauma. Additionally, the predominantly female work environment hinders male nurses from seeking social support and emotional aid after adverse events (Huang et al. 2022), indirectly intensifying their physical and mental trauma and affecting their job stability. A recent scoping review (Neves et al. 2025) further highlights gender differences in the second victim phenomenon, showing that male and female healthcare workers may experience different patterns of emotional impact and support needs. These findings underscore the importance of developing gender‐sensitive support strategies, particularly in nursing, where the workforce is predominantly female but the number of male nurses is growing. Given the growing demand for male nurses in healthcare, nursing leaders must prioritize the well‐being and mental health of male nurses, provide multiple support channels and resources, reduce male nurse turnover and stabilize the nursing workforce.

### 4.4 Attention to Young and Junior Nurses as Second Victims

The study finds that nurses with shorter work experience and younger age exhibit more intense second‐victim reactions and higher levels of professional distress. This indicates that young and junior nurses are more susceptible to the trauma of being second victims. This phenomenon can be explained by the Conservation of Resources theory (Hobfoll 1989). Junior nurses often lack accumulated psychological resources, such as coping strategies and professional confidence, to withstand trauma and high workloads further deplete these resources. As Hobfoll et al. (2018) stated, this depletion creates a vicious cycle of stress and burnout, exacerbating the suffering of young nurses as second victims after experiencing adverse events. Ajri‐Khameslou et al. (Ajri‐Khameslou et al. 2017) note that nurses with less experience are more prone to anxiety, affecting their personal lives. Young nurses, due to their shorter work experience and unstable relationships, receive less support from colleagues and management. After adverse events, due to their lack of clinical experience, young nurses may lack the ability to handle situations effectively, perceive issues in a limited manner, and lack understanding of the second victim phenomenon, leading to a decline in professional identity and the onset of burnout (Chen et al. 2020; Anfang et al. 2022; Houqin et al. 2023). If not adequately supported, this can increase their intention to leave the profession (Mok et al. 2020). Therefore, nursing leaders should focus on the harm caused by adverse events to nurses, pay more attention to young and junior nurses, establish comprehensive training systems, strengthen education on the second victim phenomenon, help them recognize and understand the second victim experience, and provide timely psychological counselling and support to restore their professional self‐efficacy, thereby reducing the turnover of young nurses and ensuring the development of nursing and patient safety.

### 4.5 Impact of Nursing Department Adverse Event Discussions

Guiru, Huimin, Rongrong, Shihua, et al. (2019) highlight that nurses who participate in discussions at the nursing department level after experiencing adverse events report higher levels of distress. The reason lies in the fact that managers often focus on the event itself, overlooking its negative impact on nurses. During the analysis and discussion of adverse events, second victims repeatedly relive the event, leading to heightened negative emotions and physical and mental distress. Conversely, a study (Zuokun et al. 2023) indicates that nurses who have participated in nursing department discussions exhibit lower psychological distress levels. This is mainly due to managers considering the complexity of clinical practice, helping second victims confront the adverse outcomes, summarize experiences and provide psychological comfort, thus alleviating their psychological stress. Currently, the impact of nursing department‐level adverse event discussions on the distress levels of second victims is a subject of controversy. Most scholars advocate that discussions on adverse events should prioritize the mental health of second victims (Tamburri 2017; Pettker 2017), as punitive and accountability measures can exacerbate nurses' distress. Given that the approach to handling adverse events varies among the hospitals conducting these studies, further research is needed to confirm the factors influencing the impact of adverse event discussions at the nursing department level.

### 4.6 Limitations

This scoping review acknowledges several potential limitations. First, the absence of a systematic quality assessment for included studies may compromise the strength of evidence. Second, publication bias may exist due to reliance on published Chinese and English literature in public databases, potentially leading to underrepresentation of studies with negative or neutral results. Although search comprehensiveness was enhanced by expanding database coverage and adhering to PRISMA‐ScR guidelines, limitations persist. Future research could further mitigate bias by registering study protocols and expanding grey literature searches. Additionally, the majority of included studies were from Asian regions, which may introduce bias into conclusions and obscure the impact of cultural differences on second victim experiences. Finally, the review predominantly included female nurses, which reflects the gender composition of the nursing workforce; however, this limits the direct generalizability of findings to male nurses, highlighting the need for future studies with more balanced gender samples. Notably, this study did not deeply explore the interactions among influencing factors, and the lack of investigation into these interrelationships—critical for a comprehensive understanding of nurses' second‐victim experiences and support needs—represents a significant limitation.

## 5 Conclusions

This scoping review found that nurses experiencing an adverse event had high levels of second‐victim distress, the most severe psychological distress and the lowest levels of perceived organizational support for nurses. The level of nurses' experience and support as second victims was influenced by sociodemographic, work, psychological and adverse event factors. The Second Victim Experience and Support Scale was found to be a valuable tool for the effective quantification and assessment of nurses' psychological distress and emotional trauma as second victims after experiencing an adverse event. It was also found to be useful in the identification of nurses' support needs after experiencing an adverse event and in the analysis of the factors affecting nurses' second victim experience and support levels. The scale is important for improving the quality of nursing care, ensuring patient safety and promoting nurses' psychological health.

To address the gaps identified in this review and to further enhance the understanding of nurses' experiences and support needs as second victims, as well as to provide effective interventions, future research could focus on the following directions:

1. Longitudinal Study Designs: Employ longitudinal research designs to track the psychological recovery trajectories of nurses following adverse events, clarifying the dynamic impacts of various factors. Utilize mixed‐methods approaches to deeply explore implicit influencing factors such as gender and culture, and conduct cross‐cultural comparisons to validate the generalizability of research findings.
2. Multicultural and Gender‐Diverse Studies: Prioritize multicultural and gender‐diverse designs in future research, expanding samples from Asia to include nurses from Western countries (e.g., the United States, Europe), Africa and Latin America, to investigate how healthcare systems influence the experiences of second victims.
3. Focused Studies on High‐Risk Groups: Conduct mixed‐methods research targeting high‐risk groups such as male nurses, novice nurses and those working in emergency/ICU departments to deeply explore their unique needs. Investigate the unique barriers that male nurses face in seeking support due to gender stereotypes or cultural stigma, and develop targeted interventions.
4. Non‐Punitive Reporting Systems and Psychological Safety Training: Promote the establishment of non‐punitive reporting systems in hospitals and incorporate psychological safety training into the evaluation of nursing managers to foster an organizational culture of ‘patient safety–nurse psychological safety’ co‐development.
5. Exploring Interactions Among Factors: Consider using statistical or qualitative methods to explore the interactions among sociodemographic, work‐related, psychological and adverse event‐related factors to understand how these factors interact and provide valuable insights into the complex experiences of nurses as second victims.

By focusing on these directions, future research can further refine the support system for nurses who are second victims, providing evidence for the development of interventions to support nurses who have experienced adverse events, thereby improving nurses' mental health and enhancing patient safety and quality of care.

## 6 Implications for Practice and Research

Understanding and addressing the phenomenon of second victims in nursing is crucial for the well‐being of nurses and their capacity to provide continuous, safe and high‐quality patient care. It is necessary to adjust the psychological state of second victims and guide them to adopt positive strategies in response to adverse events. Therefore, nursing managers should pay attention to the distress and support situation of nurses as second victims in adverse events, formulate targeted organizational support programmes for different influencing factors and different groups of nurses, and actively establish corresponding support systems to alleviate the negative impact of adverse events on nursing staff, improve nursing quality, safeguard patients' safety and promote sustainable and healthy development of medical care.

## Author Contributions

X.Y. conceived the research design, conducted the search and wrote the paper. Jingru Z., X.Y. and F.Y. participated in the identification of search terms and the development of the search strategy. X.W., K.Z. and Jiquan Z. were responsible for the selection of studies for inclusion and the extraction of data. F.Y., X.Z. and H.L. provided guidance throughout the study and proofread the English language. All authors read and approved the final manuscript.

## Funding

This study received funding from the Sichuan Medical Law Research Center project, namely YF24‐Q21 and Sichuan Applied Psychology Research Center project, namely CSXL‐26215.

## Ethics Statement

The authors have nothing to report.

## Conflicts of Interest

The authors declare no conflicts of interest.

## Supporting information


**Data S1:** nop270556‐sup‐0001‐Supinfo1.docx.

## Data Availability

Data sharing is not applicable to this article as no datasets were generated or analyzed during the current study. This is a scoping review and entirely theoretical research.
