# Supplementary material for: Factors Influencing Nurses' Experiences and Support as Second Victims Following Adverse Events: A Scoping Review
Source: Nurs Open. 2026 Aug 13;13(8):e70556. doi: 10.1002/nop2.70556 (PMC13472543; doi:10.1002/nop2.70556)
Supplement: Supplementary file 1 [file NOP2-13-e70556-s001.docx]

**Full search strategies for each database**

**PubMed**

((“nurs*”[Title/Abstract] OR “nurs* personnel”[Title/Abstract] OR “registered nurs*”[Title/Abstract] OR “health personnel”[MeSH Terms] OR “health care provider”[Title/Abstract] OR “health care professional”[Title/Abstract] OR “nurs* practitioner”[Title/Abstract])) AND ((“second victim”[Title/Abstract] OR “second victim* syndrome”[Title/Abstract] OR “second victim* phenomenon”[Title/Abstract]))

**Embase**

(‘nurs*’:ab,ti OR ‘nurs* personnel’:ab,ti OR ‘registered nurs*’:ab,ti OR ‘health personnel’:ab,ti OR ‘health care provider’:ab,ti OR ‘health care professional’:ab,ti OR ‘nurs* practitioner’:ab,ti) AND (‘second victim’:ab,ti OR ‘second victim* syndrome’:ab,ti OR ‘second victim* phenomenon’:ab,ti)

**Web of Science**

TS=(nurs* OR “nurs* personnel” OR “registered nurs*” OR “health personnel” OR “health care provider” OR “health care professional” OR “nurs* practitioner”) AND TS=(“second victim” OR “second victim* syndrome” OR “second victim* phenomenon”)

**CINAHL**

(TI (nurs* OR “nurs* personnel” OR “registered nurs” OR “health personnel” OR “health care provider” OR “health care professional” OR “nurs* practitioner”) OR AB (nurs* OR “nurs* personnel” OR “registered nurs*” OR “health personnel” OR “health care provider” OR “health care professional” OR “nurs* practitioner”)) AND (TI (“second victim” OR “second victim* syndrome” OR “second victim* phenomenon”) OR AB (“second victim” OR “second victim* syndrome” OR “second victim* phenomenon”))

**Scopus**

TITLE-ABS-KEY(nurs* OR “nurs* personnel” OR “registered nurs*” OR “health personnel” OR “health care provider” OR “health care professional” OR “nurs* practitioner”) AND TITLE-ABS-KEY(“second victim” OR “second victim* syndrome” OR “second victim* phenomenon”)

**Cochrane Library**

(nurs* OR “nurs* personnel” OR “registered nurs*” OR “health personnel” OR “health care provider” OR “health care professional” OR “nurs* practitioner”) AND (“second victim” OR “second victim* syndrome” OR “second victim* phenomenon”)

**CNKI**

(SU=nurse OR SU=nursing personnel OR SU=registered nurse) AND (SU=second victim OR SU=second victim syndrome OR SU=second victim phenomenon)

**Wanfang**

(Topic: nurse OR Topic: nursing personnel OR Topic: registered nurse) AND (Topic: second victim OR Topic: second victim syndrome OR Topic: second victim phenomenon)

**VIP**

(M=nurse OR M=nursing personnel OR M=registered nurse) AND (M=second victim OR M=second victim syndrome OR M=second victim phenomenon)

**CBM**

(nurse OR nursing personnel OR registered nurse) AND (second victim OR second victim syndrome OR second victim phenomenon)
